# *Interleukin 10* mutant zebrafish have an enhanced *interferon gamma* response and improved survival against a *Mycobacterium marinum* infection

**DOI:** 10.1038/s41598-018-28511-w

**Published:** 2018-07-09

**Authors:** Sanna-Kaisa E. Harjula, Markus J. T. Ojanen, Sinja Taavitsainen, Matti Nykter, Mika Rämet

**Affiliations:** 1 Laboratory of Experimental Immunology, BioMediTech Institute and Faculty of Medicine and Life Sciences, University of Tampere, Tampere, Finland; 2 Laboratory of Immunoregulation, BioMediTech Institute and Faculty of Medicine and Life Sciences, University of Tampere, Tampere, Finland; 3 Laboratory of Computational Biology, BioMediTech Institute and Faculty of Medicine and Life Sciences, University of Tampere, Tampere, Finland; 4 Department of Pediatrics, Tampere University Hospital, Tampere, Finland; 5 Department of Children and Adolescents, Oulu University Hospital, Oulu, Finland; 6 PEDEGO Research Unit and Medical Research Center Oulu, University of Oulu, Oulu, Finland

## Abstract

Tuberculosis ranks as one of the world’s deadliest infectious diseases causing more than a million casualties annually. IL10 inhibits the function of Th1 type cells, and IL10 deficiency has been associated with an improved resistance against *Mycobacterium tuberculosis* infection in a mouse model. Here, we utilized *M. marinum* infection in the zebrafish (*Danio rerio*) as a model for studying Il10 in the host response against mycobacteria. Unchallenged, nonsense *il10*^*e46/e46*^ mutant zebrafish were fertile and phenotypically normal. Following a chronic mycobacterial infection, *il10*^*e46/e46*^ mutants showed enhanced survival compared to the controls. This was associated with an increased expression of the Th cell marker *cd4-1* and a shift towards a Th1 type immune response, which was demonstrated by the upregulated expression of *tbx21* and *ifng1*, as well as the down-regulation of *gata3*. In addition, at 8 weeks post infection *il10*^*e46/e46*^ mutant zebrafish had reduced expression levels of proinflammatory cytokines *tnfb* and *il1b*, presumably indicating slower progress of the infection. Altogether, our data show that Il10 can weaken the immune defense against *M. marinum* infection in zebrafish by restricting *ifng1* response. Importantly, our findings support the relevance of *M. marinum* infection in zebrafish as a model for tuberculosis.

## Introduction

Annually more than 10 million new tuberculosis cases are estimated to emerge, leading to over a million casualties^1^. The immune defense against the pathogen which causes tuberculosis, *Mycobacterium tuberculosis*, requires the elaborate collaboration of both the innate and adaptive immunity, as is demonstrated by the increased disease susceptibility in recipients of TNF-antagonist as well as in HIV-positive individuals^1–3^. Accordingly, deficient, but also excessive, macrophage mediated TNF production as well as the lack of T helper (Th) 1 type cell responses compromise the host’s ability to resist a mycobacterial infection and accelerate the disease pathogenesis^4–7^.

IL10 is an anti-inflammatory cytokine that was originally identified as a protein secreted by Th2 cells able to inhibit cytokine production in Th1 cells^8,9^. Later, it was discovered that several other cell types, including both immune and nonimmune cells, produce IL10^10^. Genome-wide association studies in humans have linked *IL10* polymorphisms to susceptibility and resistance towards tuberculosis, although the results vary depending on the polymorphism studied and the study subjects^11,12^. Furthermore, *in vivo* mouse studies have shown that IL10 impairs the immune defense against *M. tuberculosis* by impeding host immunity at an early^13,14^, but also during later stages of an infection^15^. In these studies, the lack of functional IL10 signaling resulted in enhanced protection against mycobacteria and was attributed to an increased Th1 response^13–16^. As a consequence, for example, macrophages were more capable of presenting *M. tuberculosis* antigens and recruiting inflammatory cells^13,14^. Enhanced protection was demonstrated by a lower bacterial burden in the lungs and spleen^13–15^ and improved survival of the mice^15^. While IL10 deficiency or receptor blockade has been associated with an enhanced protection against mycobacteria in mice, it has also been reported that the lack of IL10 can eventually lead to harmful lung inflammation and to the progression of a mycobacterial disease in the mouse model^16^.

The zebrafish is a small teleost, which is constantly gaining popularity as a model organism. The cellular components of the zebrafish immune system, such as mononuclear phagocytes^17^, dendritic cells^18^, T cells and B cells^19–23^ and eosinophils^24^ have been described and they resemble those of humans. To date, specific transcription factors expressed by different zebrafish Th as well as Treg cells have also been identified and characterized^25,26^. Previously, lymphocyte marker gene expression has been used to characterize immune response in adult zebrafish^27^ and for example *foxp3a* has been validated as a Treg marker^28^. As for the humoral components of the zebrafish immune system, the mammalian homologs of the complement system^29^, as well as the immunoglobulin isotypes IgD, IgM and the bony fish specific immunoglobulin Z/T^30^ have been found. Overall, the zebrafish is a suitable model for immunological research (reviewed in^31^).

*Mycobacterium marinum* is a natural pathogen of the zebrafish and a close relative of *Mycobacterium tuberculosis*^32^. Comparably to *M. tuberculosis*, *M. marinum* infects macrophages^33,34^ and eventually causes a systemic disease in zebrafish, which shares pathological and histological features with human tuberculosis^35–37^. A *M. marinum* infection model in zebrafish larvae has been widely used to study the innate immune response in a mycobacterial infection, and it enables the real-time visualization and rapid screening of potential tuberculosis drugs^38–40^. Furthermore, the *M. marinum* infection model in adult zebrafish allows studying the adaptive response^35–37^.

Zebrafish *il10* has a mammalian-like gene organization and conserved IL10 signature motif ^41,42^. Furthermore, Grayfer and Belosevic^43^ have found IL10 receptor 1 in zebrafish and in goldfish (*Carassius Auratus* L.). Among their analyses, an alignment of these protein sequences with those of other vertebrates, the expression measurements in different tissues and immune cell populations at mRNA level and *in vitro* binding studies of recombinant goldfish IL10 receptor 1 and IL10 proteins, indicated conservation of the IL10 system throughout evolution. In order to study the role of Il10 in the immune defense against mycobacteria, we have here characterized an *il10*^*e46/e46*^ mutant zebrafish strain in relation to a *M. marinum* infection. Also, we aim to gain more information about *M. marinum* infection in zebrafish as a model for human tuberculosis.

## Results

### A nonsense *il10*^*e46*^ mutation creates an early stop codon in the zebrafish *il10* gene

**Figure 1:**
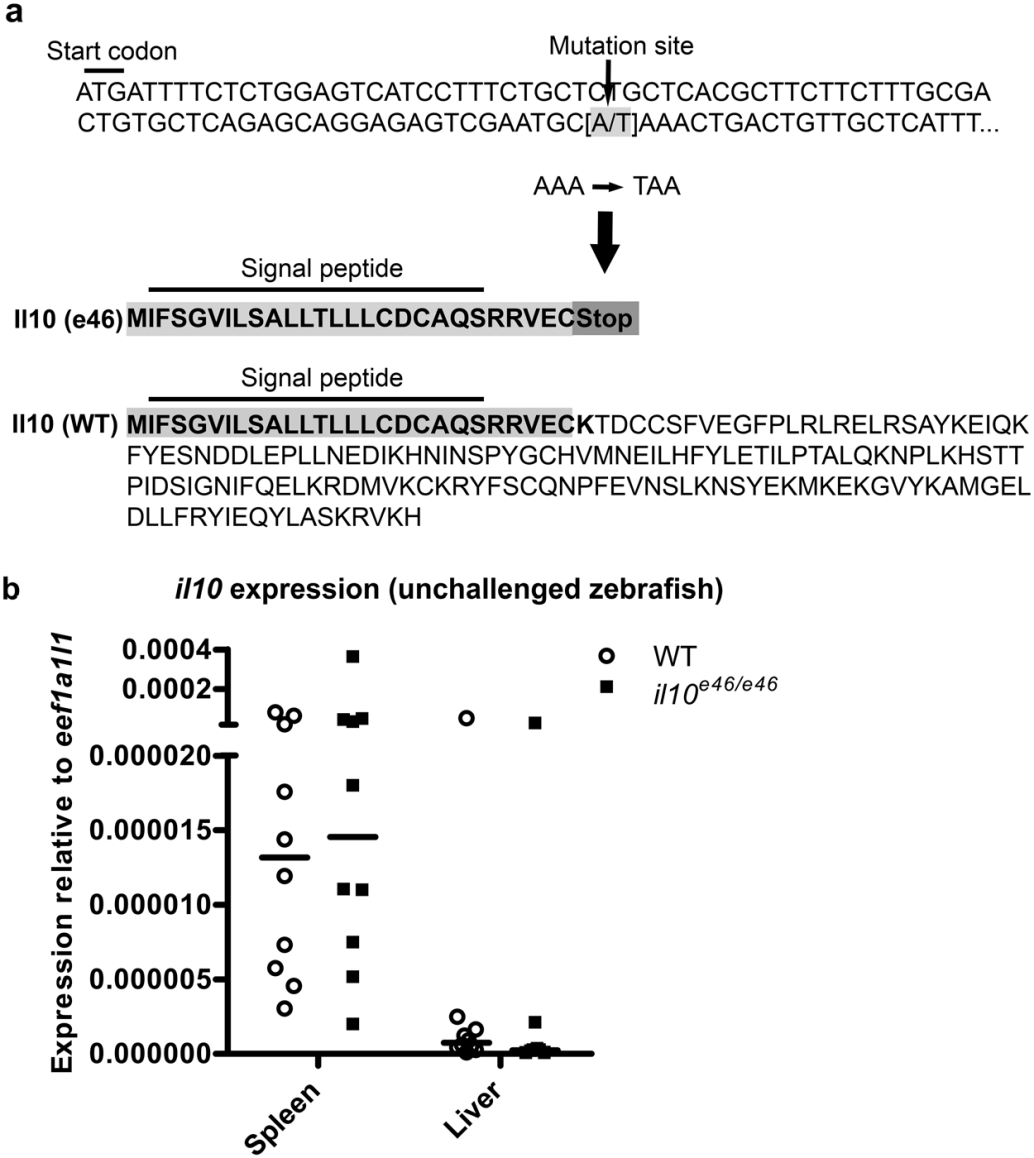
*il10*^*e46*^ point mutation in the exon 1 of the *il10* results in a premature stop codon but does not alter *il10* expression. (**a**) A schematic presentation of the *il10*^*e46*^ mutation; a adenosine (A) to thymidine (T) point mutation results in a stop codon (TAA) after the first 27 amino acids in exon 1. (**b**) Relative *il10* expression was measured with qPCR in unchallenged *il10*^*e46/e46*^ mutant and WT zebrafish, spleen and liver from the same individuals (*n* = 10 in both groups) and is presented as a scatter dot blot and median. Note the divided *y* axis. Gene expressions were normalized to the expression of *eef1a1l1*. A two-tailed Mann-Whitney test was used for the statistical comparison of differences between *il10*^*e46/e46*^ zebrafish and WT controls.

In order to study Il10 in the host response against mycobacteria the zebrafish line e46, carrying a nonsense *il10* mutation, was obtained from the Wellcome Trust Sanger Institute^44^. In the *il10*^*e46*^ mutant zebrafish a specific adenosine (A) to thymidine (T) point mutation results in a stop codon (TAA) after the first 27 amino acids in the translated region of exon 1 (Fig. 1a). As nonsense mediated decay degrades mRNA molecules producing non-functional proteins^45^, we first studied if the *il10*^*e46*^ mutation affects the levels of the *il10* mRNA, and quantified the expression of *il10* in different organs of the abdominal cavity by quantitative PCR (qPCR) (Fig. 1b). However, in any of the studied tissues *il10* mRNA expression did not differ between *il10*^*e46/e46*^ mutants and wild type (WT) zebrafish, suggesting that the effects of the mutation are only evident at the translational level. In fact, signal peptide prediction using SignalP 4.1 Server^46^ revealed that only five amino acids remain in the truncated protein, which consequently prevents the normal function of Il10 in the fish carrying the homozygous *il10*^*e46*^ mutation (Fig. 1a).

### Unchallenged *il10*^*e46/e46*^ zebrafish are phenotypically normal and have similar blood cell populations and cytokine expression profiles compared to WT fish

**Figure 2:**
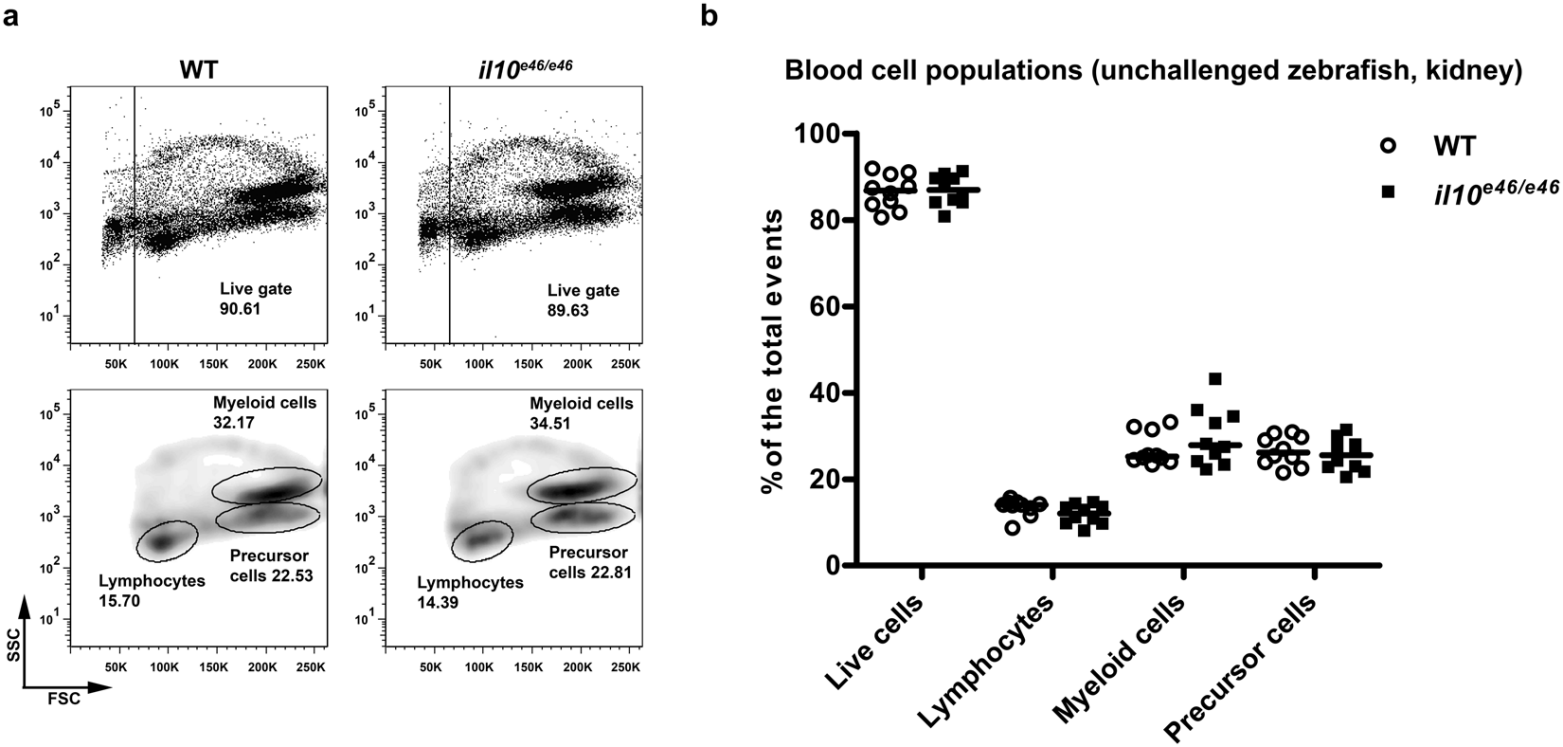
Unchallenged *il10*^*e46/e46*^ zebrafish have kidney blood cell populations similar to those of WT control fish. (**a**,**b**) The relative proportions of live cells, lymphocytes, myeloid cells and precursor cells were determined with flow cytometry in *il10*^*e46/e46*^ mutant zebrafish and in WT fish (*n* = 10 in both groups) based on granularity (SSC) and cell size (FSC). Representative flow cytometry plots are shown in panel (a). Gated populations are outlined, and the cell counts inside the gates are given as the percentages of the total viable cell population. The median of the relative proportions of different blood cell populations is presented as a scatter plot in panel (b). A two-tailed Mann-Whitney test was used for the statistical comparison of differences between *il10*^*e46/e46*^ zebrafish and WT controls.

IL10 knock-out (KO) mice have growth defects and suffer from chronic intestinal inflammation leading to 30% mortality before 3 months of age^47,48^. Like IL10 KO mouse strains, *il10*^*e46/e46*^ zebrafish are fertile and can be maintained by spawning homozygous mutant siblings^47^. However, in contrast to mice, *il10*^*e46/e46*^ mutant fish are phenotypically normal and do not have increased mortality compared to WT zebrafish. Our flow-cytometric analysis of the blood cell populations in kidney blood cell isolates revealed no differences in live cell, lymphocyte, myeloid cell or blood cell precursor cell counts in unchallenged *il10*^*e46/e46*^ zebrafish compared to the WT control fish (Fig. 2a,b).

Colitis in IL10 KO mice is attributed to the increased production of inflammatory mediators such as TNF and IL1b as well as to a hyper-activated Th1 response^47,49,50^. In order to study signs of inflammation and T cell homeostasis in unchallenged *il10*^*e46/e46*^ zebrafish, we extracted RNA from different adult zebrafish tissues and measured the expression levels of selected proinflammatory cytokines (*il1b*, *tnfa* and *tnfb)*, Th cell cytokines *(ifng1* and *il4*) as well as T cell markers *cd4-1* and *cd8a)* and a B cell marker *IgM* (Supplementary Fig. S1). In contrast to the IL10 KO mice, qPCR analysis from the liver and spleen revealed no differences in the relative expression levels of the studied inflammatory markers *tnfa*, *tnfb* and *il1b* between *il10*^*e46/e46*^ and WT fish. The expression levels of the Th1 cytokine *ifng1* and the Th2 cytokine *il4* were also comparable between the fish groups. In addition, no differences in the mRNA expression of the T cell marker genes *cd8a* and *cd4-1*, between *il10*^*e46/e46*^ and WT fish, were observed. The expression of *IgM* in the liver of *il10*^*e46/e46*^ fish was decreased compared to controls (*P* = 0.029). Collectively, these data indicate that the *il10*^*e46/e46*^ mutant zebrafish have no apparent immune abnormalities under unchallenged conditions.

In order to study in more detail the possible inflammation in the intestine of the Il10 deficient zebrafish, we collected the intestine from 1-year-old WT and *il10*^*e46/e46*^ fish and measured the expression levels of several inflammatory cytokines and immune cell markers (Supplementary Fig. S2a). No difference in the expression of the inflammatory cytokines (*il1b, tnfa* and *tnfb)*, T lymphocyte markers (*cd4-1* and *cd8a)*, B lymphocyte marker *IgM*, Th2 and Treg markers, *(gata3* and *foxp3a*, respectively), or in Th1 and Th2 hallmark cytokines (*ifng1* and *il4*, or in *il10)* was observed between WT and *il10*^*e46/e46*^ fish. The expression of Th1 cell marker *tbx21* was higher in WT fish compared to *il10*^*e46/e46*^ mutants (*P* = 0.023) but this is unlikely a sign towards colitis in *il10*^*e46/e46*^ mutants. Furthermore, the visual appearance of WT and *il10*^*e46/e46*^ intestine was similar with identifiable intestinal bulb, mid-intestine and posterior-intestine (Supplementary Fig. S2b).

### Survival of adult *il10*^*e46/e46*^ mutants is improved in a low-dose *M. marinum* infection

**Figure 3:**
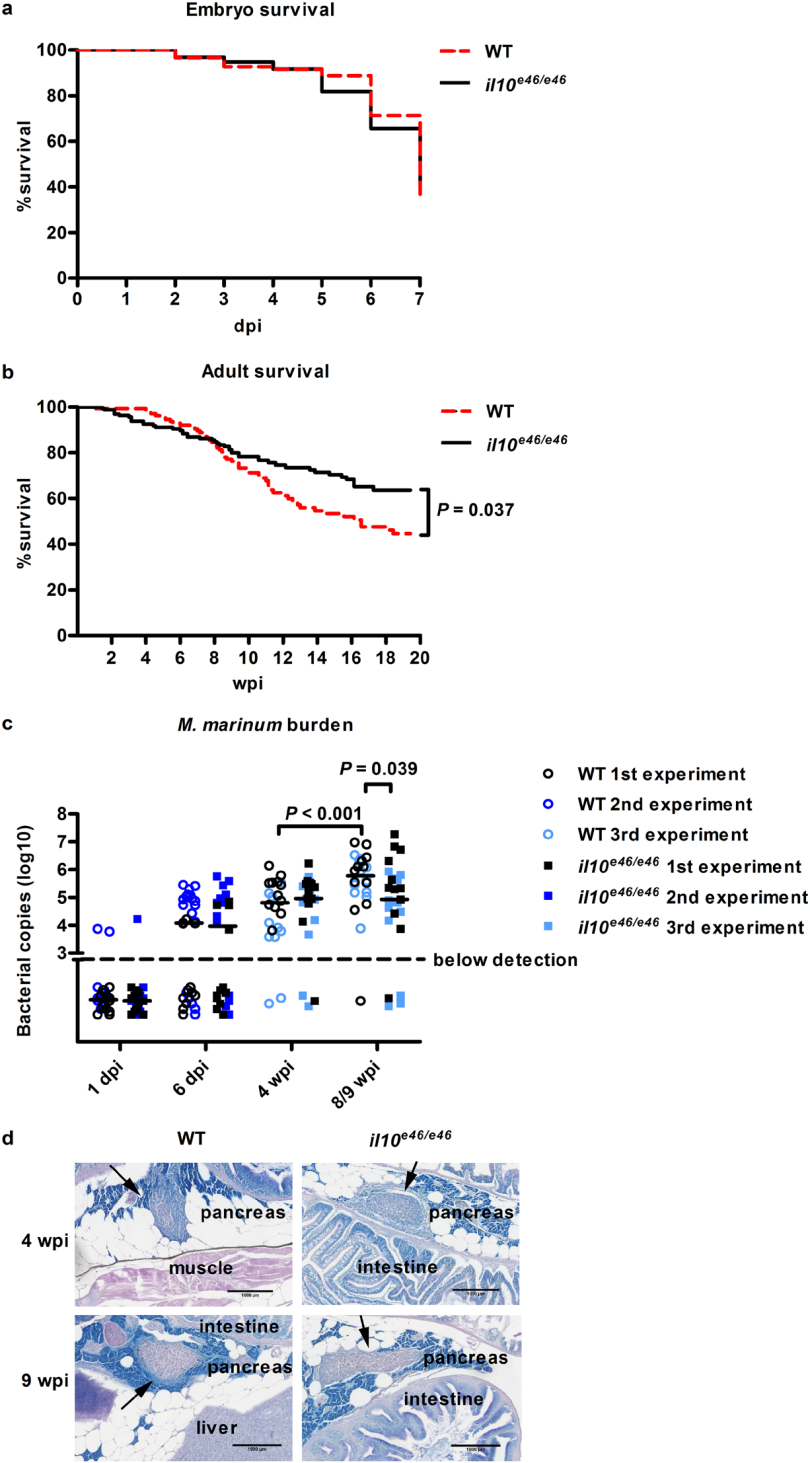
Adult *il10*^*e46/e46*^ zebrafish have enhanced survival compared to WT controls in a low-dose *M. marinum* infection. (**a**) *il10*^*e46/e46*^ (*n* = 158) and WT (*n* = 181) zebrafish larvae were microinjected before 6 hours post fertilization with *M. marinum* (3–29 CFU) and their survival was monitored for 7 days. The experiment was done six times and the data presented here is collected from one representative experiment. (**b**) The survival of adult *il10*^*e46/e46*^ (*n* = 172) and WT (*n* = 149) zebrafish was monitored for 16–20 weeks after a low-dose (2–156 CFU) mycobacterial infection. The data were collected from four experiments. (**c**) The *M. marinum* burden in abdominal organ blocks (including kidney in the first and the second experiment, without kidney in the third experiment) of adult *il10*^*e46/e46*^ mutant zebrafish (*n* = 22–27) and WT controls (*n* = 22–30 fish) was quantified with qPCR at 4 and 8/9 weeks post a low-dose infection (1–18 CFU). The bacterial load is presented as a scatter dot plot and as the median of total bacterial copies (log_10_). The data were collected from three experiments. (**d**) *M. marinum* granulomas were detected with Ziehl-Neelsen staining (*n* = 4 in both groups at both time points) at 4 and 9 weeks post a low-dose infection (2–9 CFU). Representative individuals from each group are shown. Granulomas are indicated with arrows. For panels (a) and (b) a log-rank (Mantel-Cox) and for panel (c) A two-tailed Mann-Whitney test was used for the statistical comparison of differences.

In mice, the absence of functional IL10 signaling leads to the improved control of a *M. tuberculosis* infection, and consequently, to better survival^13,15^. Other studies have, however, associated IL10 deficiency to increased inflammation leading to reduced survival^16^. To study the role of Il10 in the immune defense against a *M. marinum* infection in zebrafish, we first infected *il10*^*e46/e46*^ and WT embryos into the yolk at 0–6 hours post fertilization and followed the survival of the larvae for seven days (Fig. 3a). After three days, mortality was observed in both of the groups resulting in ca. 70% mortality at the end of the follow-up with no significant difference between *il10*^*e46/e46*^ and WT larvae.

Next, we injected a low-dose of *M. marinum* into the abdominal cavity of adult *il10*^*e46/e46*^ and WT zebrafish and followed the survival of the fish for 20 weeks (Fig. 3b). During the first 8 weeks, we observed ca. 20% mortality in both of the fish groups, which was similar to our previously reported results from a low-dose mycobacterial infection^35,51^. However, from week eight onwards the mortality of the WT fish increased compared to the *il10*^*e46/e46*^ mutants with an endpoint survival of 44% and 63%, respectively (*P* = 0.037).

In order to differentiate between resistance and tolerance against a *M. marinum* infection, we collected abdominal organ block samples from infected fish during the course of a mycobacterial infection. The samples were taken at 1 day post infection (dpi), 6 dpi, 4 weeks post infection (wpi) and 8/9 wpi, and bacterial amounts were analyzed. In our qPCR based bacterial quantification, detectable amounts of *M. marinum* in the infected zebrafish were primarily observed for the first time at the 6 dpi time point (Fig. 3c). At 6 dpi, but also at 4 wpi, no differences in *M. marinum* counts between *il10*^*e46/e46*^ mutants and WT zebrafish were observed (at 6 dpi; bacterial copy number median 65,000 vs 74,000, at 4 wpi; median 126,000 vs 90,000, Fig. 3c). However, at 8/9wpi there was a smaller bacterial amount in the *il10*^*e46/e46*^ fish compared to the WT fish (median 86,000 vs 645,000, *P* = 0.039). In addition, bacterial counts in *il10*^*e46/e46*^ fish remained stable between 4 and 8/9 weeks, whereas there was a clear increase in bacteria in WT fish (median 65,000 vs 645,000, *P* < 0.001) (Fig. 3c). A Ziehl-Neelsen staining of formalin fixed and paraffin embedded zebrafish confirmed the presence of mycobacteria in both infected *il10*^*e46/e46*^ and WT fish at 4 and 9 wpi (Fig. 3d). The histopathological analysis did not reveal any differences in granuloma morphology or in their tissue distribution (Supplementary Fig. S3). In conclusion *il10*^*e46/e46*^ mutant zebrafish showed improved survival but no apparent changes in histopathology. In addition, there was a decrease in bacterial burden of *il10*^*e46/e46*^ mutants at 8/9 wpi fish indicating enhanced resistance rather than a higher tolerance against low dose *M. marinum* infection.

### *il10*^*e46/e46*^ mutant zebrafish have an enhanced *ifng1* response in a low-dose mycobacterial infection

**Figure 4:**
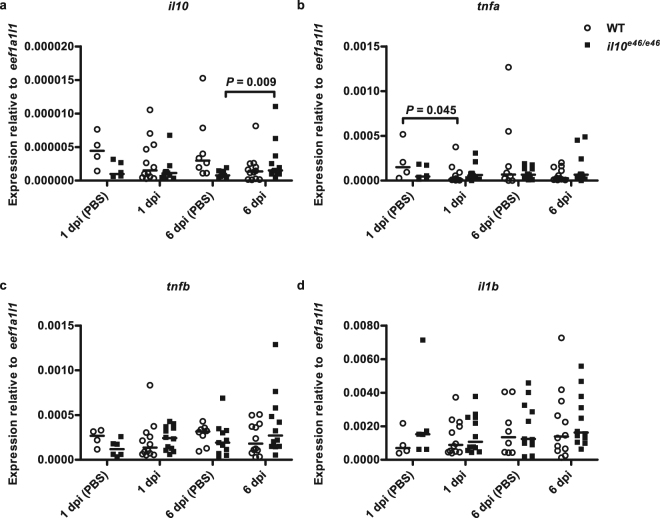
Nonfunctional *il10* does not increase the expression of proinflammatory cytokines at the early stages of a low-dose (2–9 CFU) mycobacterial infection in zebrafish. (**a–d**) The relative expression levels of *il10* and proinflammatory cytokine genes (*il1b*, *tnfa* and *tnfb*) were measured in the abdominal organ blocks (including kidney) of *il10*^*e46/e46*^ mutant fish (*n* = 6–12) and WT controls (*n* = 5–12) at 1 dpi and 6 dpi and are presented as a scatter dot plot and median. Note the different scales of the *y* axes. Gene expressions were normalized to the expression of *eef1a1l1*. The data were collected from a single experiment. A two-tailed Mann-Whitney test was used for the statistical comparison of differences.

**Figure 5:**
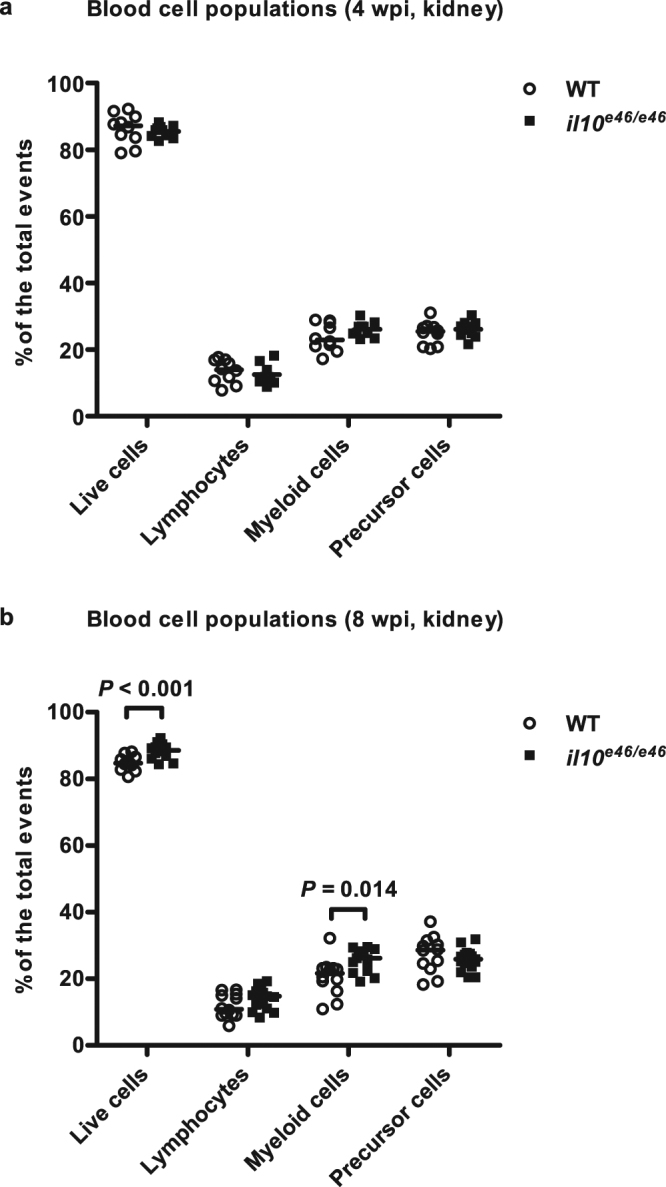
*il10*^*e46/e46*^ mutation associates with a higher proportion of myeloid cells at 8 weeks post a low-dose (1–18 CFU) *M. marinum* infection. (**a**,**b**) The relative proportions of live cells, lymphocytes, precursor cells and myeloid cells were determined with flow cytometry from the kidneys of *il10*^*e46/e46*^ mutants (*n* = 10–15) and WT control fish (*n* = 10–12) at 4 and 8 wpi based on granularity (SSC) and cell size (FSC). The data were collected from a single experiment and are presented as a scatter dot plot and median. A two-tailed Mann-Whitney test was used for the statistical comparison of differences between *il10*^*e46/e46*^ zebrafish and WT controls.

**Figure 6:**
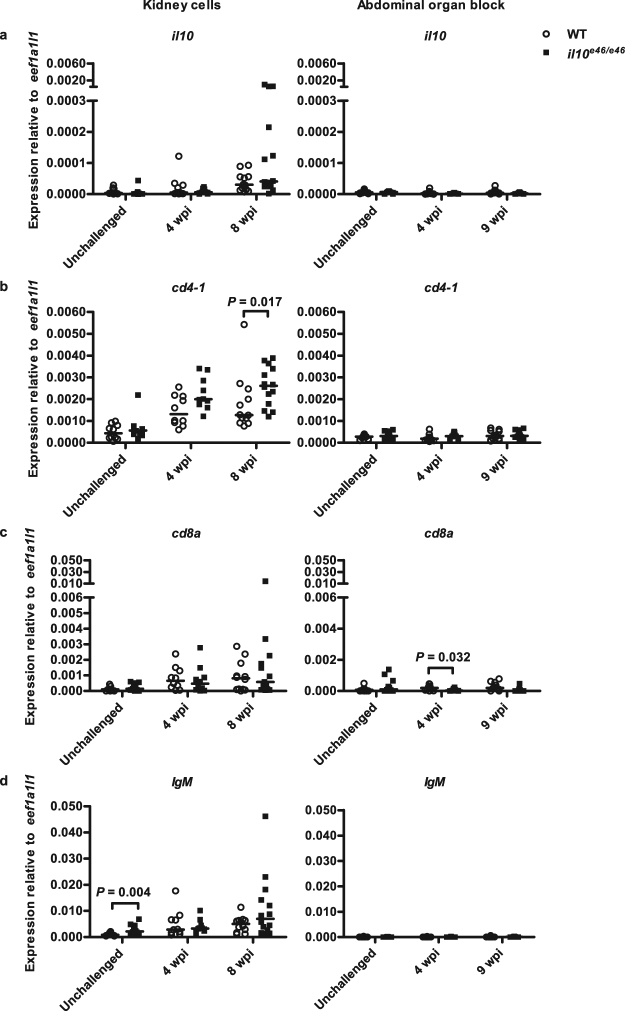
*il10*^*e46/e46*^ mutant zebrafish have an elevated Th cell marker, *cd4-1*, expression level at 8 weeks post a *M. marinum* infection. (**a**–**d**) The relative expressions of *il10* and lymphocyte markers (*cd4-1, cd8a* and *IgM*) in kidneys of unchallenged *il10*^*e46/e46*^ mutants (*n* = 9–10) and WT control fish (*n* = 9–10) and in the unsorted kidney cell populations of *il10*^*e46/e46*^ mutants (*n* = 10–14) and WT control fish (*n* = 10–12) at 4 and 8 weeks post a low-dose (1–18 CFU) infection (on the left) as well as in the abdominal organ blocks (including kidney) of unchallenged *il10*^*e46/e46*^ mutants (*n* = 12) and WT control fish (*n* = 11–12) and at 4 and 9 weeks post a low-dose (2–9 CFU) infection (*n* = 9–12 and *n* = 11–12, respectively) (on the right) were measured with qPCR. Data are presented as a scatter dot plot and median. Note the different scales of the *y* axes and the divided *y* axis in panels a and c. Gene expressions were normalized to the expression of *eef1a1l1*. Each dataset was collected from a single experiment. A two-tailed Mann-Whitney was used for the statistical comparison of differences.

**Figure 7:**
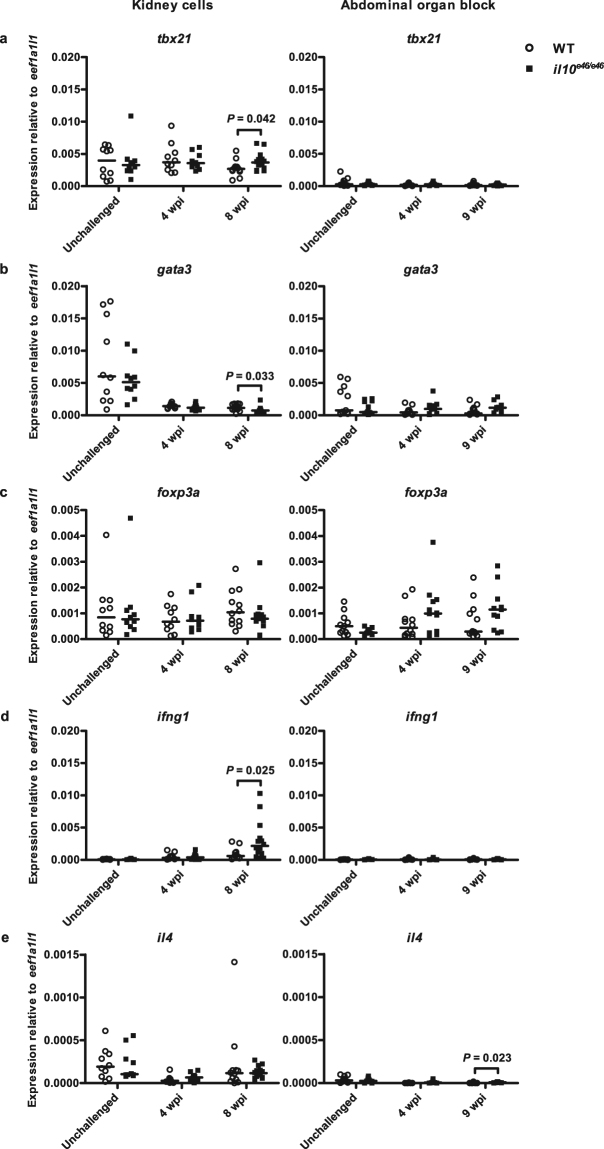
*il10*^*e46/e46*^ mutant zebrafish have an enhanced Th1 cell mediated immune response in a mycobacterial infection. (**a**–**e**) The relative expressions of Cd4 + lymphocyte transcription factors (*tbx21*, *gata3*, *foxp3a*) and Th cell cytokines (*ifng1* and *il4*) in kidneys of unchallenged *il10*^*e46/e46*^ mutants (*n* = 10) and WT control fish (*n* = 10) and in the unsorted kidney cell populations of *il10*^*e46/e46*^ mutants (*n* = 10–14) and WT control fish (*n* = 10–12) at 4 and 8 weeks post a low-dose (1–18 CFU) infection (on the left) as well as in the abdominal organ blocks (including kidney) of unchallenged *il10*^*e46/e46*^ mutants (*n* = 12) and WT control fish (*n* = 11–12) and at 4 and 9 weeks post a low-dose (2–9 CFU) infection (*n* = 12 and *n* = 12, respectively) (on the right) were measured with qPCR. Data are presented as a scatter dot plot and median. Gene expressions were normalized to the expression of *eef1a1l1*. Each dataset was collected from a single experiment. A two-tailed Mann-Whitney was used for the statistical comparison of differences.

**Figure 8:**
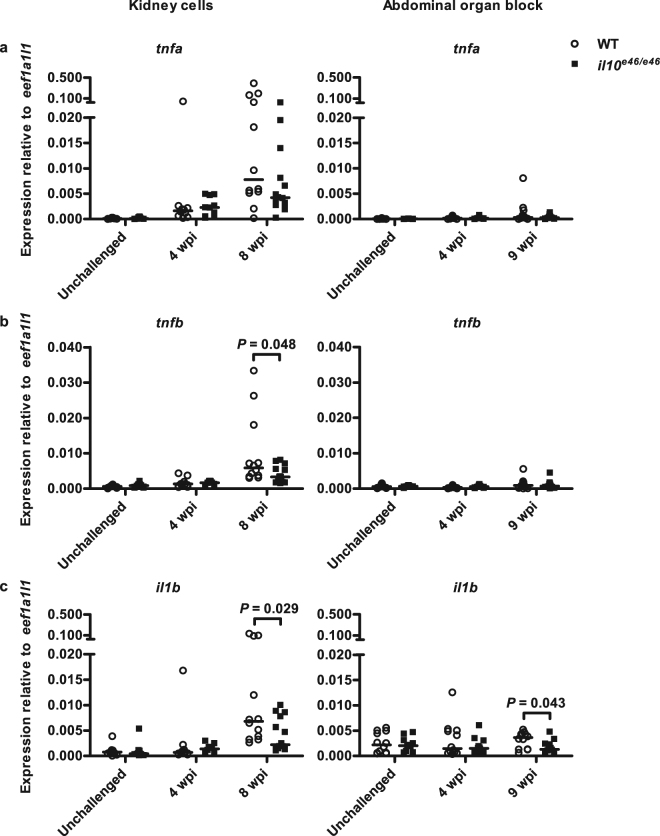
*il10*^*e46/e46*^ mutant zebrafish have reduced inflammation in a chronic mycobacterial infection. (**a**–**c**) The relative expression of selected proinflammatory cytokines (*il1b*, *tnfa*, *tnfb*) in kidneys of unchallenged *il10*^*e46/e46*^ mutants (*n* = 10) and WT control fish (*n* = 10) and in the unsorted kidney cell populations of *il10*^*e46/e46*^ mutants (*n* = 10–14) and WT control fish (*n* = 10–12) at 4 and 8 weeks post a low-dose (1–18 CFU) infection (on the left) as well as in the abdominal organ blocks (including kidney) of unchallenged *il10*^*e46/e46*^ mutants (*n* = 12) and WT control fish (*n* = 12) and at 4 and 9 weeks post a low-dose (2–9 CFU) infection (*n* = 12 and *n* = 12, respectively) (on the right) were measured with qPCR. Data are presented as a scatter dot plot and median. Note the different scales of the *y* axes and the divided *y* axis in panel a. Gene expressions were normalized to the expression of *eef1a1l1*. Each dataset was collected from a single experiment. A two-tailed Mann-Whitney was used for the statistical comparison of differences.

To study the mechanisms underlying enhanced survival in *il10*^*e46/e46*^ mutant zebrafish against a low-dose *M. marinum* infection we quantified the expression of selected cytokines and immune cell markers and conducted a flow cytometric analysis in the zebrafish kidney blood cells at different time points post infection (Figs 4–8). The expression levels of the studied genes in the kidney and abdominal organ block samples of unchallenged WT and *il10*^*e46/e46*^ fish were similar. However, the relative *IgM* expression in the *il10*^*e46/e46*^ mutant kidney was increased 2.5-fold compared to WT zebrafish (*P* = 0.004) but otherwise the mutants did not show any differences compared to WT.

First, we quantified the expression of *il10, tnfa*, *tnfb*, and *il1b* in the abdominal organ blocks of zebrafish at early time points during an infection (Fig. 4). Until 6 dpi, *M. marinum* infection did not alter the expression of *tnfb* or *il1b* either in *il10*^*e46/e46*^ mutants or in WT fish compared to corresponding PBS injected controls. However, similarly to previous reports about the upregulation of *Il10* upon immunogenic stimulation^52,53^, *il10* was significantly upregulated in the *il10*^*e46/e46*^ mutants at 6 dpi in comparison to PBS controls (*P* = 0.009). WT fish, in turn, had a slight reduction in *tnfa* expression at 1 dpi compared to PBS injected fish (*P* = 0.045). As in unchallenged zebrafish, we did not see any differences in the expression levels of the cytokine genes between the *il10*^*e46/e46*^ mutants and WT fish at 1 dpi or 6 dpi. At these early time points, expression levels of the T cell markers *cd4-1* (CD4 + cells) or *cd8a* (CD8 + cells) or the B cell marker *IgM* did not differ between *il10*^*e46/e46*^ and WT fish either (Supplementary Fig. S4). These data indicate that a low-dose mycobacterial infection does not cause acute systemic inflammation in zebrafish and that the *il10*^*e46/e46*^ zebrafish have a transcriptional innate cytokine response similar to the WT controls in the low-dose infection.

In order to study the role of Il10 later in a *M. marinum* infection, we conducted a flow cytometric analysis in the zebrafish kidney blood cells at 4 and 8 wpi and analyzed the relative amounts of lymphocyte, precursor cell and myeloid cell populations in *il10*^*e46/e46*^ mutants and WT controls (Fig. 5). There were no differences in the relative lymphocyte or precursor cell counts between the groups. However, the relative proportion of myeloid cells was significantly lower in WT fish compared to the *il10*^*e46/e46*^ mutants (median 21.6% vs. 26.2%, *P* = 0.014). Of note, the total live cell numbers were also lower in the WT controls compared to *il10*^*e46/e46*^ mutant fish at 8 wpi (*P* < 0.001). Furthermore, in the WT control group the median of the relative myeloid cell count was 14.5% lower compared to the median of unchallenged fish at 8 wpi (*P* = 0.001).

To obtain more quantitative data on T and B lymphocytes in *il10*^*e46/e46*^ mutant and WT fish at 4 and 8 wpi, we next extracted RNA from the unsorted kidney cell samples used for the flow cytometry and determined relative expression levels of *il10*, the T cell markers *cd4-1* and *cd8a* as well as the B cell marker *IgM* (Fig. 6). No differences in the relative *il10* expression levels between the *il10*^*e46/e46*^ mutant and the WT groups were observed. Interestingly, a qPCR analysis showed approximately 34.3% higher relative expression of *cd4-1* in *il10*^*e46/e46*^ mutants compared to WT control fish at 4wpi (*P* = 0.052, NS) and a significantly higher expression at 8 wpi (*P* = 0.017). No differences in the kidney blood cell *cd8a* nor the B lymphocyte marker *IgM* expression were detected between *il10*^*e46/e46*^ and WT zebrafish at 4 and 8 wpi. Notably, however, *cd8a* expression was significantly lower in *il10*^*e46/e46*^ abdominal organ blocks compared to WT controls at 4 wpi (*P* = 0.032) (Fig. 6). Abdominal organ blocks did not have statistically significant differences in their *cd4-1* or *IgM* expression levels between *il10*^*e46/e46*^ mutants and WT fish, although a trend towards the upregulation of *cd4-1* was seen at 4 wpi (*P* = 0.053, NS, Fig. 6). Altogether, the upregulated *cd4-1* expression in the *il10*^*e46/e46*^ mutant kidneys is a possible consequence of an enhanced Th cell response in the infected *il10*^*e46/e46*^ zebrafish.

In addition to the effects of *Il10* in regulating the production of proinflammatory cytokines in mice, human *IL10* is known to suppress the activation of Th cells by inhibiting the production of IFNG and IL4^54^. Hence, we quantified the relative expression of the Th1, Th2 and Treg cell transcription factors, *tbx21*, *gata3* and *foxp3a*, respectively, as well as the canonical Th1 and Th2 cell cytokine genes *ifng1* and *il4* in the kidney blood cells (Fig. 7). Indicative of an enhanced Th1 type immune response, our qPCR analysis showed that *il10*^*e46/e46*^ mutant fish had higher relative expression levels of *tbx21* at 8wpi (*P* = 0.042), whereas the expression of *gata3* was lower compared to WT controls at the same time point (*P* = 0.033). Additionally, expression of the Th1 type cytokine gene *ifng1* was upregulated in *il10*^*e46/e46*^ fish compared to WT zebrafish 8 wpi (*P* = 0.025). The expression of the canonical Th2 cytokine gene *il4* was instead similar in both *il10*^*e46/e46*^ and WT fish the unsorted kidney cells at 4 and 8 wpi. Nor were any differences seen in the expression levels of *foxp3a* between the groups at either of the time points, suggesting a similar transcriptional Treg cell response in both mutants and WT fish during an infection. Mutation in *il10* can also cause differential tissue and cell type specific regulation of Th type gene expression since upregulation of *il4* was observed in the abdominal organ blocks of the *il10*^*e46/e46*^ mutants compared to WT zebrafish at 9 wpi (*P* = 0.023, Fig. 7).

A mycobacterial infection elicits the host’s immune cells to produce humoral effectors such as complement components, reactive oxygen and nitrogen intermediates as well as proinflammatory cytokines to fight the infection^55^. In general, the magnitude of this response can be used to assess the severity of the prevalent bacterial disease. To compare the expression of mediators of inflammation in *il10*^*e46/e46*^ mutants and WT fish in a chronic *M. marinum* infection, we quantified the expression of *tnfa*, *tnfb* and *il1b* in the kidney blood cells at 4 and 8 wpi (Fig. 8). qPCR results showed that *il10*^*e46/e46*^ mutants had significantly lower expression levels of *tnfb* (*P* = 0.048) and *il1b* (*P* = 0.029) compared to WT fish at 8 wpi. No differences were observed in the expression of *tnfa* between *il10*^*e46/e46*^ mutants and WT fish. Similar results were seen in the abdominal organ blocks as the relative expression of *il1b* was downregulated in *il10*^*e46/e46*^ mutant fish compared to WT controls (*P* = 0.043, Fig. 8). Together, the elevated *tbx21* and *ifng1* expressions as well as the reduced expression of *gata3* in the *il10*^*e46/e46*^ mutants indicate that a nonsense mutation in *il10* leads to a Th1 cell type immune response in zebrafish. In addition, this Th1 cell response associates with lower *tnfb* and *il1b* expression presumably indicating the slower progress of a mycobacterial infection as the expression of these cytokines have been shown to associate with the bacterial burden during the reactivation of *M. marinum* infection^56^.

**Figure 9:**
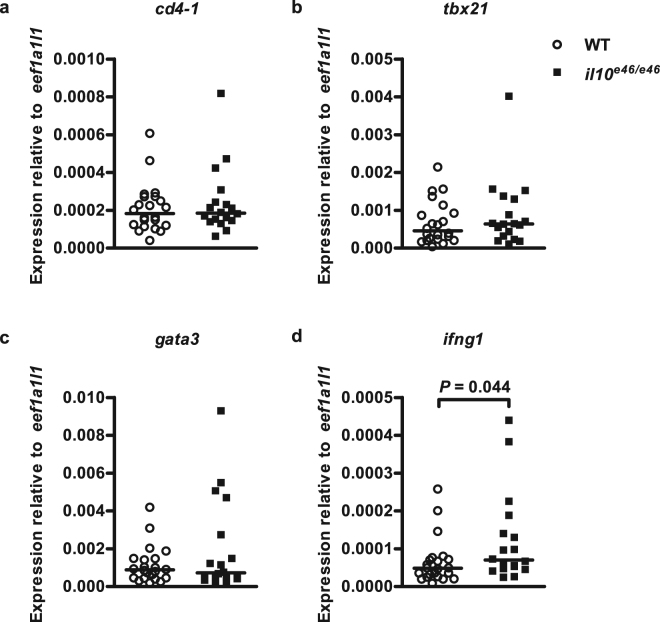
*il10*^*e46/e46*^ mutant progeny of *il10*^*e46/*+^ zebrafish have an elevated *ifng1* expression level in a *M. marinum* infection. (**a**–**d**) *il10*^*e46/e46*^ mutants were outcrossed to wild type AB zebrafish and their heterozygous progeny was incrossed. The ungenotyped offspring of *il10*^*e46/+*^ fish was infected with a low-dose (8–17 CFU) of *M. marinum*. The relative expression of lymphocyte marker (*cd4-1*), Cd4 + lymphocyte transcription factors (*tbx21*, *gata3*) and Th1 cytokine (*ifng1*) was measured in the kidneys of *il10*^*e46/e46*^ (*n* = 18) and WT (*n* = 22) zebrafish at 8 wpi. Note the different scales of the *y* axes. Gene expressions were normalized to the expression of *eef1a1l1*. The data were collected from a single experiment. A two-tailed Mann-Whitney was used for the statistical comparison of differences.

The *il10*^*e46/e46*^ mutants are produced by ENU mutagenesis and thus likely contain also other mutations in their background in addition to the *e46* mutation. To address this, we outcrossed *il10*^*e46/e46*^ mutants to wild type AB zebrafish and incrossed their heterozygous progeny. Thereafter, we infected ungenotyped offspring of the heterozygous *il10*^*e46/*+^ mutants with a low-dose of *M. marinum* and collected zebrafish kidneys for gene expression analysis (*cd4-1*, *tbx21*, *gata3 and ifng1*) 8 wpi. Also in this setting, *ifng1* expression was enhanced in *il10*^*e46/e46*^ compared to controls (Fig. 9). We did not see significant differences in the expression levels of *cd4-1*, *tbx21* or *gata3*. However, the elevated *ifng1* expression further supports the notion that a nonsense mutation in *il10* leads to a Th1 cell type immune response in zebrafish.

To further evaluate if there are significant co-segregating mutations, we extracted the DNA from the abdominal organ blocks of the offspring of the heterozygous *il10*^*e46/+*^ mutants and performed whole genome sequencing. We compared the sequence of the coding regions of *il10*^*e46/e46*^ mutants and WT fish to the GRCz11 reference genome and to the sequence of WT AB zebrafish. The putative mutations found in the analysis are shown in the Supplementary Table S1. 57 mutations with a mutant allele fraction equal to or greater than 25% caused either a stop codon or a frameshift (Supplementary Table S2). One of these mutant alleles in gene *si:dkey-19a16.2* had a 100% allele fraction in both *il10*^*e46/e46*^ mutants and WT zebrafish indicating that it is homozygous in both. The allele fraction of 17 mutations shown in the Supplementary Table S2 differed significantly (*P* < 0.05) between *il10*^*e46/e46*^ and WT zebrafish (Supplementary Table S3). As expected, *e46* allele had 100% fraction in *il10*^*e46/e46*^ sample and 0% fraction in WT sample. Noteworthy, there were no other mutations that would cause either a nonsense or a frameshift mutation in the chromosome 11 (Supplementary Table S2). This indicates that none of these types of mutations are likely to be co-segregating with the *e46* mutation. The role of the other detected mutations in the immunity or other phenotypes remains to be evaluated.

## Discussion

The exact mechanisms of the immune defense against *M. tuberculosis* are in many ways still unknown, although disease progression has been studied in human samples, as well as with various model organisms *in vivo* (reviewed in^57^). Although spontaneous latency occurs in *M. tuberculosis* infected maqaque^58^ and rabbit^59,60^, these models raise serious ethical concerns. On the other hand, a *M. tuberculosis* infection in mouse is a well-established model for tuberculosis, but accomplishing a latent disease requires either a prior Bacillus Calmette-Guérin vaccination to boost the host’s immune response or a period of antibiotic treatment post infection in order to prevent an acute infection^61^. To study mycobacterial pathogenesis in the context of a natural host-pathogen interaction, a *M. marinum* infection in several fish species such as medaka^62^, goldfish^43,63,64^ and zebrafish^35–37^ is used to model tuberculosis (reviewed in^65^). Compared to other fish models, the zebrafish *M. marinum* infection has its benefits due to fast disease progression as well as the small size of the host^43,62–64^. In addition, a mycobacterial infection in adult zebrafish spontaneously reaches a latent state that can be reactivated with an immuno-suppressive treatment^35^.

IL10 KO mice develop an age-related enterocolitis, demonstrated by symptoms such as weight loss, diarrhea and abnormal gut morphology^50^. More specifically, the lack of IL10 leads to the excess production of proinflammatory cytokines, such as IL1b and TNF, as well as increased Th1 activation leading to the over-production of IFNG^47,50^. Interestingly, unchallenged *il10*^*e46/e46*^ zebrafish did not show any detectable signs of auto-immunity before the age of 10 months; the maximum age of the zebrafish used in the study. This is in line with the observed similar expression levels of the proinflammatory cytokine genes *tnfa*, *tnfb* and *il1b*, as well as the hallmark Th1 cytokine gene *ifng1* in all of the studied tissues in *il10*^*e46/e46*^ mutants compared to WT control fish. Furthermore, no difference was observed in the relative expression levels of the T cell markers *cd8a* and *cd4-1* or in our flow cytometric analysis of lymphocyte counts. In addition, Treg cells have been shown to be required in maintaining peripheral tolerance to prevent autoimmunity^66–68^ and recently, Sugimoto *et al*. have studied zebrafish colitis by using *foxp3a* mutant zebrafish^28^. While they found the inflammatory phenotype in these mutants, it did not lead to death as quickly as the corresponding genotype in mice. Consequently, they suggested that this may be due to the milder immune cell effector responses in aquatic vertebrates, including fish, which in turn may contribute to the ability of scarless regeneration of fish. Moreover, also in fish the microbes of the environment affect commensal bacteria, which in turn can explain the differences in enterocolitis between mice and fish^69^. It has also been suggested that fish maintenance in constant water circulation systems (similar to ones we have used) can prevent development of intestinal inflammation to some extent^70^. Noteworthy, in our studies, *foxp3a* expression was not altered in *il10*^*e46/e46*^ mutants. Since IL10 has been shown to maintain the *FOXP3* expression in Treg cells in mice and humans, it points to a compensatory mechanism in fish^71^. Furthermore, Brugman *et al*. have speculated that there could be other immune mediators in addition to *il1b* and *il10* which contribute to the development of enterocolitis in zebrafish^70^. Of note, we did observe increased *IgM* expression in the kidney as well as decreased *IgM* expression in the liver of *il10*^*e46/e46*^ zebrafish. Increased expression is in line with the previously reported higher serum immunoglobulin levels in IL10 deficient mice^48,50^ whereas decreased expression of *IgM* in the liver supports studies demonstrating carp IL10 in enhancing IgM + B cell proliferation^72^. Together these results indicate that, in contrast to IL10 KO mice, unchallenged *il10*^*e46/e46*^ mutant zebrafish have neither an overt inflammatory phenotype nor an inherently over-activated T cell response.

IL10 has been widely studied for its potent role in tuberculosis. Interestingly, mouse models of tuberculosis have not only reported that the lack of IL10 is beneficial to the host in a mycobacterial infection, but also that an IL10 deficiency can have detrimental effects on immunological control. For example, CBA/J background mice, which are susceptible to tuberculosis and whose IL10 function has been blocked, as well as *Il10*^−/−^ mice, survived longer than control mice^14,15^, whereas *Il10*^−/−^ mice with the C57BL/6 J background were all dead after ca. 27 weeks, while all the WT controls survived^16^. Among other things, these contradictory results have been attributed to differences in mouse and bacterial strains and to variation between mouse phenotypes (e.g. gut flora)^73^. Here, we first used zebrafish embryos in the context of a mycobacterial infection to specifically study the innate immune response prior to the development of the adaptive immunity. In our embryonic *M. marinum* infection, the mortality of *il10*^*e46/e46*^ mutant zebrafish larvae did not differ significantly from the WT controls. This suggests that in our model a nonsense mutation in the *il10* gene does not improve, or compromise, resistance against mycobacteria during the early innate immune response. Accordingly, mouse models of tuberculosis have indicated that IL10 deficient mice show no mortality until ca. 14 wpi^16^ indicating a significant adaptive immune component in infection control. Using different injection route might lead to different outcome since the bacteria is able to proliferate in the yolk sac before it spreads into the different tissues^74^. However, Carvalho *et al*. have showed that the infection of *M. marinum* into the yolk sac leads to the formation of initial stages of granulomas, similar to ones that form when the caudal vein infection route is used^38^. The yolk sac infection method was also validated by showing that the decrease in the myeloid cell count lead to increased, and use of antibiotics to decreased *M. marinum* burden in the larvae^38^. Thus, since yolk sac infection is time-effective, it was chosen for the current study.

Next, we infected adult zebrafish with a low-dose of *M. marinum* and followed the fish for an average of 18.5 weeks. Curiously, the mortality of adult *il10*^*e46/e46*^ zebrafish was on average significantly lower compared to their WT controls. The impaired survival of WT zebrafish compared to *il10*^*e46/e46*^ mutants may be due to their inability to reach latency or a higher tendency for spontaneous reactivation. In fact, the function of IL10 has been linked to the reactivation of a chronic pulmonary *M. tuberculosis* infection in transgenic mice, which produce increased amounts of IL10^75^. In the study by Turner *et al*., the over-production of IL10 associated with a significantly increased bacterial burden as well as with decreased expression of *Tnf* and *Il12p40* as signs of reactivation. Mechanistically, the higher susceptibility to mycobacterial reactivation in WT fish could be a consequence of an unfavorable inflammatory TNF/IL10 balance in the tuberculous granuloma as was previously reported *in silico*^76^.

In our low-dose infection model, we have previously demonstrated that a proinflammatory phenotype leading to an enhanced immediate immune response can associate with a decreased bacterial burden later in an infection at 9 wpi^51^. In addition, an appropriate TNF level has been shown to be important for tuberculosis immunity, as both the deficient and excessive production of TNF are linked to accelerated pathogenesis^7^. Here, we saw that, as in unchallenged zebrafish, the expression of the proinflammatory cytokine genes *tnfa*, *il1b* and *tnfb* in *il10*^*e46/e46*^ mutant fish was at the same level compared to WT controls at 1 and 6 dpi. Furthermore, at these early time points after a low-dose *M. marinum* infection, the expression of *il1b* and *tnfb* was not upregulated compared to the fish injected with PBS. This is consistent with our previous studies^35^ and with mouse studies in which TNF levels are not elevated in the serum or lungs of IL10 deficient mice before 14 and 15 dpi, respectively^13^. Expectedly, we did not see any changes or significant induction of the lymphocyte markers *cd4*, *cd8* or *IgM* at 1 or 6 dpi. This is in line with the onset of the adaptive immune response only after two weeks post the mycobacterial infection^55,77^.

The immune response against tuberculosis requires an interplay between several of the host’s immune cells, the most important being macrophages, dendritic cells and CD4 + T lymphocytes^78^. Our flow cytometric analyses at 4 and 8 wpi exhibited similar lymphocyte amounts in *il10*^*e46/e46*^ mutant zebrafish compared to WT controls. Interestingly, however, *il10*^*e46/e46*^ mutant fish had significantly higher relative amounts of kidney myeloid cells compared to WT zebrafish at 8 wpi and further studies are warranted to understand the downstream effects of the lack of Il10 leading to increased myeloid cell counts after a mycobacterial infection. Although we could not detect any differences in total lymphocyte amounts between *il10*^*e46/e46*^ and WT zebrafish, our qPCR analysis on the unsorted kidney cell samples showed a trend of elevated *cd4-1* expression in *il10*^*e46/e46*^ mutant fish compared to WT controls at 4 wpi and a significantly higher expression at 8 wpi. This may also be due to some other cell types than lymphocytes, such as dendritic cells and macrophages. However, this result is similar to IL10 deficient mice, in which elevated CD4 + T cell amounts are observed at different time points after an infection, and may imply an enhanced Th response in *il10*^*e46/e46*^ mutants^14,15^.

Th1 cells are important in attacking intracellular pathogens such as *M. tuberculosis*^4–6^. In tuberculosis, IFNG produced by Th1 cells activates macrophages, which results in the stimulation of phagocytosis, phagosome maturation, the production of reactive nitrogen intermediates and antigen presentation^79^. Furthermore, a *M. tuberculosis* infection in *Il10* deficient mice has also been shown to lead to the enhanced production of IFNG by T cells^13–15^. *il10*^*e46/e46*^ mutant zebrafish had enhanced expression levels of the Th1 marker, *tbx21*, and decreased expression levels of the Th2 marker, *gata3*, at 8wpi. In addition, the Th1 cytokine *ifng1* was upregulated at 8 wpi in *il10*^*e46/e46*^ mutants further suggesting a shift towards a Th1 cell mediated response. The upregulation of *ifng1* in *il10*^*e46/e46*^ compared to WT control fish was also seen in the siblings from a heterozygous *il10*^*e46/*+^ incross. This further confirms the enhanced production of *ifng1*, likely from Th1 cells, in the absence of Il10^14,15^. However, since the whole genome sequencing revealed also other mutant alleles causing a stop codon or frameshift in addition to the *e46* allele, we cannot exclude the possibility that these mutations contribute to our results. Noteworthy, with the allele fragment cut-off of 25% there were no stop codon or frameshift causing mutations in the chromosome 11 where *il10* is located. In other words, our analysis suggests no significant co-segregating damaging mutations.

It has been previously shown that Th2 type response (*gata3/tbx21* ratio) after four weeks of a *M. marinum* infection is associated with a low bacterial burden in the wild type AB fish^27^. In this study, adult wild type AB zebrafish were infected with a low-dose of *M. marinum* and were divided into three subgroups based on the bacterial burden at different time points. In the subgroup of the lowest bacterial burden, a Th2 type marker gene expression bias was observed. However, also Th1 response is induced (as indicated by elevated *ifng1* expression also in this current study) in response to *M. marinum* infection^35^. Thus, for the optimal immune response both Th1 and Th2 types of responses are apparently required. Our current data indicate that lack of Il10 results in enhanced *ifng1* expression and in better survival.

Although the role of IL10 in the host defense against *M. tuberculosis* is unambiguous, *in vivo* studies have concluded that in the absence of functional IL10 signaling, the Th1 response is hyper-activated resulting in improved resistance against a mycobacterial infection^13,15,73^. Consistent with this, our results show that a nonsense mutation in the zebrafish *il10* leads to improved survival after a *M. marinum* infection, and associates with an enhanced Th1 response against mycobacteria. The higher Th1/Th2 ratio in the chronically infected *il10*^*e46/e46*^ mutant zebrafish is reflected by elevated *ifng1* and *tbx21* expression levels as well as decreased *gata3* mRNA levels at 8 wpi. We did not detect differences in the expression of the proinflammatory cytokine genes *tnfa*, *il1b* and *tnfb* between unchallenged *il10*^*e46/e46*^ mutants and WT fish at the early stages of a *M. marinum* infection at 1 and 6 dpi. Taken together, these results suggest that the lack of Il10 does not enhance the early proinflammatory response after a *M. marinum* inoculate, but instead they highlight the importance of the Th1 response in resistance against a mycobacterial infection. Furthermore, our study validates the use of the zebrafish model of a *M. marinum* infection in tuberculosis studies.

## Methods

### Zebrafish lines and maintenance

Four to ten months old zebrafish were used in the adult experiments. The *il10* mutation carrying zebrafish line e46 was obtained from Wellcome Trust Sanger Institute (Hinxton UK)^44^. In addition, wild type AB zebrafish from the Tampere Zebrafish Core Facility were used in whole genome sequencing experiment. Unchallenged fish were maintained in a standard flowthrough system (Aquatic Habitats, Florida, USA) with an automated light/dark cycle of 14 h and 10 h and fed with SDS 400 food (Special Diets Services, Essex, UK) twice and with in-house cultured *Artemia nauplia* once a day. During the time when gene expression measurements in the intestine, infection experiment with outcrossed e46 line and the whole genome sequencing were done, the fish were fed with GEMMA Micro 500 food (Skretting, Stavanger, Norway) once a day. The genotypes of the fish were confirmed by Sanger sequencing performed by our faculty’s sequencing facility. Homozygous fish and wild type (WT) siblings were spawn as separate groups and maintained separately for the experiments. *M. marinum* infected fish were maintained in a standard flowthrough system (Aqua Schwarz GMbH, Göttingen, Germany) in the aforementioned light/dark cycle and fed with SDS 400 food twice a day. Infected fish were monitored daily and humane endpoint criteria defined in animal experiment permits were applied. The Animal Experiment Board of Finland has approved the zebrafish housing, care, and all of the experiments (permits ESAVI/10079/04.10.06/2015, ESAVI/10823/04.10.07/2016 and ESAVI/2464/04.10.07/2017). The same ethical regulations as for all other vertebrate model animals are applied for zebrafish and all methods of this article were performed in accordance with relevant guidelines and regulations.

### qPCR

Both RNA and DNA were extracted from kidney, spleen, liver, abdominal organ blocks (+/−kidney, which is the main hematopoietic tissue in zebrafish) and kidney blood cells with TRIreagent (Molecular Research Center, Ohio, USA) following the manufacturer’s coextraction protocol. The quality of the RNA was validated from several abdominal organ block (including kidney) samples of with 1.5% agarose (Bioline, London, United Kingdom) gel electrophoresis. The RNA samples were treated with the RapidOut DNA Removal Kit (Thermo Fischer Scientific, Waltham, USA) to remove genomic DNA. For the RNA samples, reverse transcription was done with the SensiFAST^TM^ cDNA synthesis kit (BioLine, London, UK) and the relative gene expression levels of target genes were determined from cDNA with quantitative PCR (qPCR) using the PowerUp™ SYBR® master mix (Thermo Fischer Scientific). The qPCR primer sequences and the ZFIN identification codes for the analyzed genes are given in Supplementary Table S4. The expression levels of the target genes were calculated relative to the expression of *eef1a1l1*^80^ using 2^-ΔCt^ method. Total DNA was used to quantify the *M. marinum* colony forming units (CFU) with qPCR using SensiFAST™ SYBR® No-ROX (Bioline)^35^. The detection limit of this method was considered 1,000 CFU. qPCR was performed with a CFX96 qPCR machine (Bio-Rad, California, USA) and the data was analyzed with the Bio-Rad CFX Manager software v1.6 (Bio-Rad). Genomic DNA contamination was controlled by including no reverse transcriptase controls to the cDNA synthesis and to the following qPCR reaction from randomly selected RNA samples. The specificity of the qPCR for each amplified region was validated with a melt curve analysis and with a 1.5% agarose gel electrophoresis from a series of selected samples.

### Imaging of zebrafish intestine

The intestine of *il10*^*e46/e46*^ mutants and WT zebrafish was imaged with Nikon SMZ745T microscope and DS-Vi1 camera (Nikon, Minato, Tokyo, Japan). The scale bar was measured with NIS-Elements D 4.2 software (Nikon).

### Flow cytometry

Flow cytometry was performed as described previously^19,51^. In short, adult zebrafish were euthanized with 0.04% 3-amino benzoic acid ethyl ester and their kidneys were isolated and suspended in PBS with 0.5% fetal bovine serum (Gibco/Invitrogen, California, USA). Relative amounts of lymphocytes, blood cell precursors and myeloid cells in unchallenged and infected zebrafish were determined with a FACSCanto II (Becton, Dickinson, New Jersey, USA) and the data were analyzed with the FlowJo program (v7.5; Tree Star, Inc, Oregon, USA). After flow-cytometry, the remaining kidney cell suspensions from the *M. marinum* infected zebrafish were pelleted with 1,000 g for 1 min at + 4 °C, washed with 500 µl of PBS, centrifuged at 10,000 g for 1 min at + 4 °C, resuspended in 700 µl of TRIreagent (Molecular Research Center, Ohio, USA) and stored at −20 °C until RNA extraction. The remaining abdominal organ block (excluding kidney) from the infected fish were also collected and stored at −80 °C until DNA extractions. Throughout the article these remaining organs (without kidney) from flow cytometry are referred the abdominal organ block. In other experiments, abdominal organ block refers to all the organs isolated from the abdominal cavity (including kidney).

### Experimental *M. marinum* infections

*M. marinum* (ATCC 927) was cultured and inoculated as described previously^35^. However, here *M. marinum* was suspended in phosphate buffered saline (PBS) rather than in potassium chloride prior to infections. In the zebrafish embryos, PBS with 2% polyvinylpyrrolidone-40 and 0.3 mg/ml phenol red (Sigma-Aldrich, Missouri, USA) was used as a mycobacterial carrier solution. A volume of 1 nl was injected 0–6 hours post fertilization into the yolk sac with aluminosilicate capillary needles (Sutter instrument Co., California, USA) using a micromanipulator (Narishige International, London UK) and a PV830 Pneumatic PicoPump (World Precision Instruments, Sarasota, Florida, USA) and visualized with a Stemi 2000 microscope (Carl Zeiss MicroImaging GmbH, Göttingen, Germany). Survival was followed daily by inspecting the larvae under a microscope. For the adult zebrafish infections, fish were anesthetized with 0.02% 3-amino benzoic acid ethyl ester, and 5 µl of *M. marinum* with 0.3 mg/ml phenol red (Sigma-Aldrich, Missouri, USA) was injected into the abdominal cavity with a 30 gauge Omnican 100 insulin needle (Braun, Melsungen, Germany). The *M. marinum* amounts (CFU) used in both the embryonic and adult infections were verified by plating bacterial inoculates on 7H10 agar (Becton Dickinson, New Jersey, USA) plates.

### Ziehl-Neelsen staining

The presence of *M. marinum* in infected adult zebrafish was verified with Ziehl-Neelsen staining from paraffin embedded tissue sections as described previously^35,81^. Visualization of stained sections was performed using an Objective Imaging Surveyor virtual slide scanner (Objective Imaging, Cambridge, United Kingdom) and the scanned sections were digitized with a 20 × Plan Apochromatic microscope objective at a resolution of 0.4 μm/pixel. The image data were converted to JPEG2000 format as described previously^82^. The granulomas were counted and classified according to Myllymäki *et al*.^56^.

### Whole genome sequencing

The DNA samples extracted from the offspring of the heterozygous *il10*^*e46/+*^ mutants and wild type AB fish was used for the whole genome sequencing. In each sample DNA of ten fish was pooled together. The RNA removal, Kapa Hyper Plus -library preparation and the whole genome sequencing were conducted at the Institute for Molecular Medicine Finland FIMM Technology Centre, Helsinki, Finland. The DNA libraries were sequenced with the 150 bp paired-end sequencing on the Illumina NovaSeq 6000 platform with a sequencing depth of >110 gigabases/sample.

### Whole genome sequence alignment and quality control

Paired-end reads were processed prior to alignment to remove sequencing adapters and low-quality bases at the read tails. Adapters were trimmed using cutadapt-1.11^83^ in paired mode. Low-quality bases at read ends with a smoothed base quality <25 were trimmed using an in-house algorithm. After quality control, the paired-end reads were aligned against the GRCz11 zebrafish reference genome using Bowtie-2.3.0^84^. Optical and PCR duplicates were removed using samblaster-0.1.24^85^.

### Mutation analysis from the whole genome sequencing data

Mutations were called from the *il10*^*e46/e46*^ mutant and WT zebrafish using an in-house pipeline. This was done by identifying variants with an alternate allele fraction of at least 20% and at least 5 supporting reads. The allele fraction was also required to be 20 times higher than the alternate allele fraction of the mutation in the AB zebrafish. Mutations and their protein-level effects were annotated using ANNOVAR^86^. Frameshift and stop-gain mutations were curated using Integrative Genomics Viewer (IGV)^87^.

### Statistical analyses

Web-based ClinCalc program (http://clincalc.com/Stats/SampleSize.aspx) was used for all of the sample size calculations. Based on our previous adult zebrafish survival experiments with a low-dose *M. marinum* infection, we estimated the end-point mortality difference between WT and *il10*^*e46/e46*^ zebrafish to be 40%. With a 80% statistical power, a minimum group size of 22 was determined for the survival experiments. In order to estimate the bacterial quantification sample size for the current research, our previously published *M. marinum* quantification with 0.5 unit difference (log10 scale, standard deviation 0.5) between study groups^51^ was used as a guideline. Using the desired 80% power this difference accounted for a group size of 16 fish for the bacterial quantification.

Statistical analyses of the results, except the whole genome sequencing data, were performed with the Prism program, version 5.02 (GraphPad Software, Inc, California, USA). The statistical significance from the survival experiments was determined with a log-rank (Mantel-Cox) test and from the flow cytometry and qPCR experiments with a nonparametric Mann-Whitney analysis. When analyzing the whole genome sequencing data, *P* values for each mutation were calculated between the *il10*^*e46/e46*^ mutants and WT fish using Fisher’s exact test. *P* values of <0.05 were considered significant.

### Data availability

The datasets generated and analyzed during the current study are available from the corresponding author on reasonable request. The whole genome sequencing data is available at European Nucleotide Archive (https://www.ebi.ac.uk/ena/; ERP number: ERP109293).

## Electronic supplementary material

Supplementary material

Supplementary Table S1

Supplementary Table S2

Supplementary Table S3
