# Supplementary material for: Interleukin 10 mutant zebrafish have an enhanced interferon gamma response and improved survival against a Mycobacterium marinum infection
Source: Sci Rep. 2018 Jul 9;8:10360. doi: 10.1038/s41598-018-28511-w (PMC6037744; doi:10.1038/s41598-018-28511-w)
Supplement: Supplementary file 1 — Supplementary material [file 41598_2018_28511_MOESM1_ESM.pdf]

interleukin 10 mutant zebrafish have an enhanced _interferon gamma_ response and improved survival against a _Mycobacterium marinum_ infection

Sanna-Kaisa E. Harjula, Markus J.T. Ojanen, Sinja Taavitsainen, Matti Nykter, Mika Rämet*

***Corresponding author:** Correspondence to Mika Rämet, phone: 358-50-4336276, Email: mika.ramet@uta.fi

Supplementary Figures

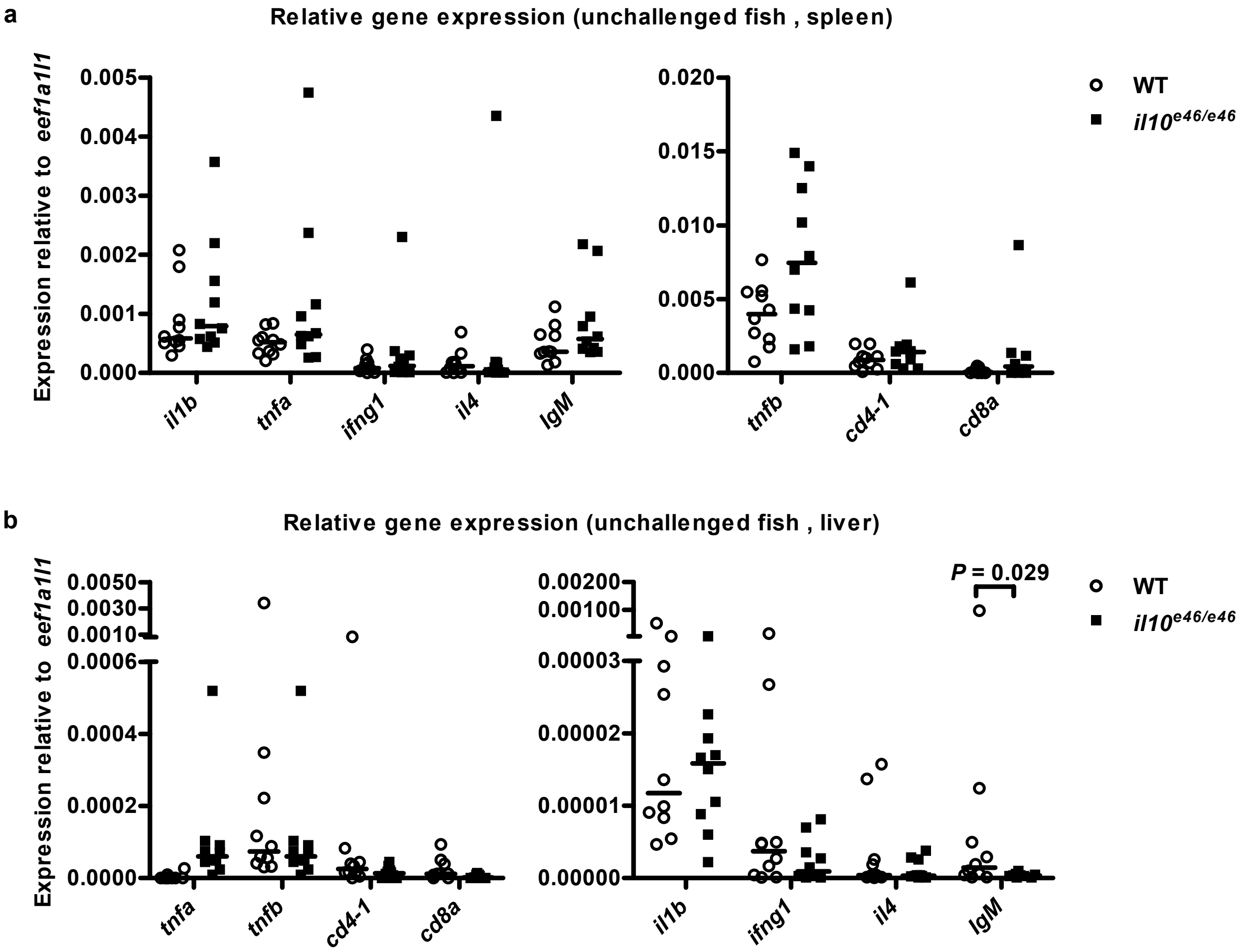

Supplementary Figure S1

Unchallenged *il10$^{e46/e46}$* zebrafish have similar expression profiles of lymphocyte markers and cytokines as WT control fish in the spleen and liver **(a - b)**. The relative expression of selected proinflammatory cytokines (*il1b*, *tnfa*, *tnfb*), Th cell cytokines *(ifng1* and *il4*), T cell markers *(cd4 and cd8)* as well as a B cell marker (*IgM*) in *il10$^{e46/e46}$* mutant zebrafish and WT controls in spleen and liver from the same individuals (*n* = 10 in both groups) were quantified with qPCR and are represented as scatter dot plot and median. Note the different scales of the *y* axes and the divided *y* axis in panel b. Gene expressions were normalized to the expression of *eef1a1l1.* A two-tailed Mann-Whitney test was used for the statistical comparison of differences between *il10$^{e46/e46}$* zebrafish and WT controls.

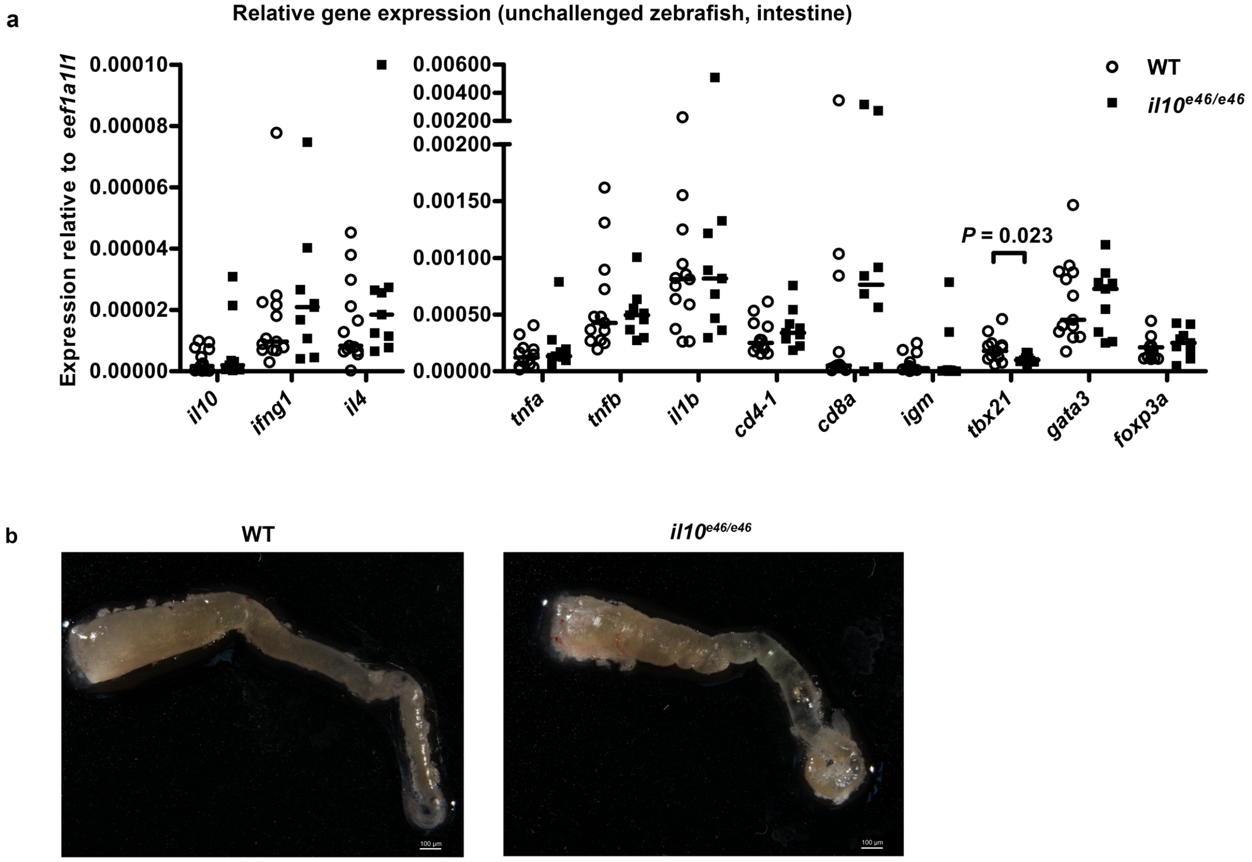

Supplementary Figure S2

Unchallenged *il10*$^{e46/e46}$ zebrafish have similar expression profiles of lymphocyte markers and cytokines as WT control fish in the intestine. **(a)** The relative expression of *il10*, selected proinflammatory cytokines (*il1b*, *tnfa*, *tnfb*), Th cell cytokines (*ifng1* and *il4*), T cell markers *(cd4-1 and cd8a*), a B cell marker (*IgM*) as well as Cd4+ lymphocyte transcription factors (*tbx21, gata3*, *foxp3a*) in *il10*$^{e46/e46}$ mutant zebrafish (n = 9) and WT controls (n = 13) in intestine were quantified with qPCR and are represented as scatter dot plot and median. Note the different scales of the y axes and the divided y axis. Gene expressions were normalized to the expression of *eef1a1l1.* A two-tailed Mann-Whitney test was used for the statistical comparison of differences between *il10*$^{e46/e46}$ zebrafish and WT controls. **(b)** The visual appearance of *il10*$^{e46/e46}$ mutant and WT control zebrafish intestine was studied by microscopy (n = 3 in both groups). A representative image from each group is shown.

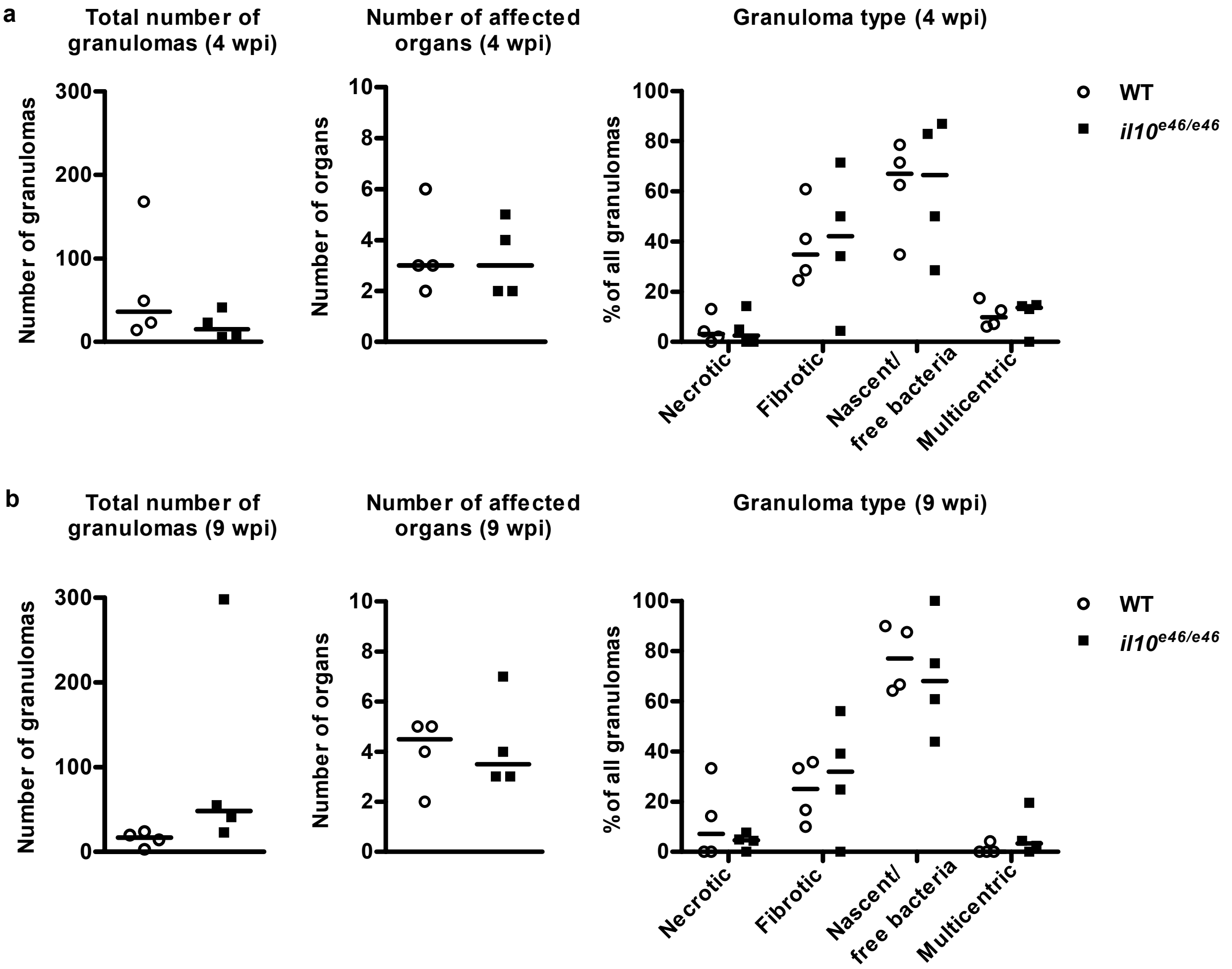

Supplementary Figure S3

There are no differences in the quantity or quality of granulomas between *il10*$^{e46/e46}$ zebrafish and WT controls in histological analysis. **(a-b)** The number of *M. marinum* granulomas were counted and the affected organs and the type of granulomas were identified in *il10*$^{e46/e46}$ zebrafish and WT controls from Ziehl-Neelsen stained sections (n = 4 in both groups at both time points) at 4 and 9 weeks post a low-dose infection (2 - 9 CFU). The granulomas were placed in one or several of the following categories: necrotic, fibrotic, multicentric, and nascent granuloma/a site of free bacteria. A two-tailed Mann-Whitney test was used for the statistical comparison of differences between *il10*$^{e46/e46}$ zebrafish and WT controls.

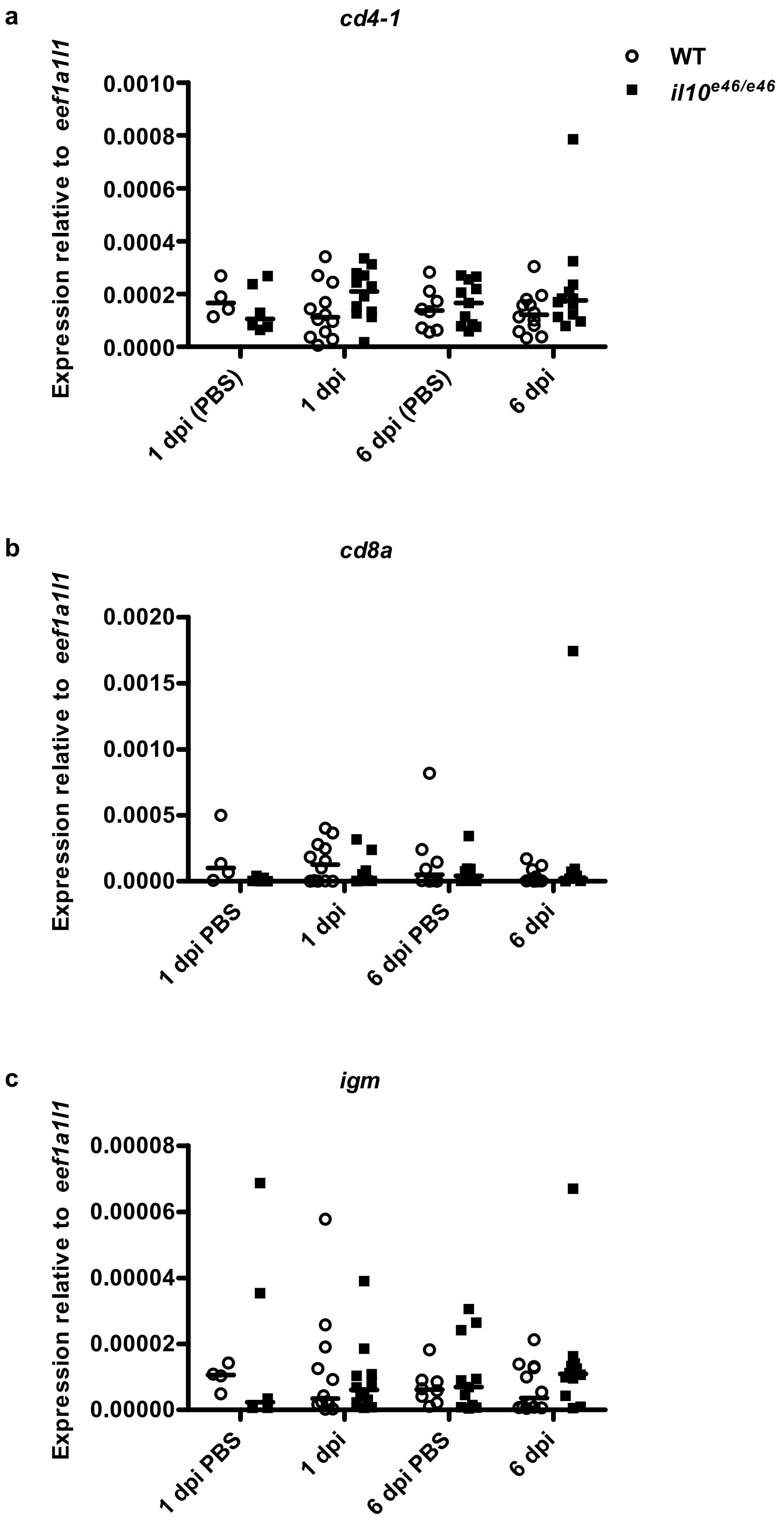
a
cd4-1
WT
il10e46/e46
Expression relative to eef1a1l1
0.0010
0.0008
0.0006
0.0004
0.0002
0.0000
1 dpi (PBS)
1 dpi
6 dpi (PBS)
6 dpi
b
cd8a
Expression relative to eef1a1l1
0.0020
0.0015
0.0010
0.0005
0.0000
1 dpi PBS
1 dpi
6 dpi PBS
6 dpi
c
igm
Expression relative to eef1a1l1
0.00008
0.00006
0.00004
0.00002
0.00000
1 dpi PBS
1 dpi
6 dpi PBS
6 dpi

Supplementary Figure S4

il10$^{e46/e46}$ mutation does not affect the expression levels of lymphocyte markers at the early stages of a low-dose mycobacterial infection. **(a - c)** The relative expression levels of T cell markers (*cd4-1* and *cd8a*) as well as the B cell marker *IgM* were measured in the abdominal organ blocks (including kidney) of *il10*$^{e46/e46}$ mutant fish (n = 4 - 12) and WT controls (n = 5 - 12) at 1 dpi and 6 days post a low-dose (2 - 9 CFU) infection and are presented as a scatter dot plot and median. Note the different scales on the y axes. Gene expressions were normalized to the expression of *eef1a1l1*. The data were collected from a single experiment. In panels a - c a two-tailed Mann-Whitney test was used for the statistical comparison of differences.

Supplementary Tables S1-S3 are provided as separate files.

Supplementary Table S1

All mutations found in the whole genome sequencing analysis. *P* values were calculated with Fisher's exact test, $P < 0.05$ was considered significant.

Supplementary Table S2

Stop codon or frameshift causing mutations found in the whole genome sequencing analysis. Mutations with allele fraction of ≥ 25 % in either of the groups are shown here. *P* values were calculated with Fisher's exact test, $P < 0.05$ was considered significant.

Supplementary Table S3

Stop codon or frameshift causing mutations found in the whole genome sequencing analysis that differ significantly between *il10*$^{e46/e46}$ and WT zebrafish. *P* values were calculated with Fisher's exact test, $P < 0.05$ was considered significant.

Supplementary Table S4

qPCR primers used for gene expression analysis

Gene[1]	ZFIN ID	Human gene ortholog (HGNC)	Sequence 5' - 3'	Reference
eef1a1l1 (ef1a)	ZDB-GENE-990415-52	*EEF1A1*	F CTGGAGGCCAGCTCAAACAT	(80)
			R ATCAAGAAGAGTAGTACCGCTAGCATTAC	
il10	ZDB-GENE-051111-1	*IL10*	F GCTCTGCTCACGCTTCTTC	(51)
			R TGGTTCCAAGTCATCGTTG	
tnfa	ZDB-GENE-050317-1	*TNF*	F GGGCAATCAACAAGATGGAAG	(35)
			R GCAGCTGATGTGCAAAGACAC	
tnfb	ZDB-GENE-050601-2	*TNF*	F GCATGTGATGAAGCCAAACG	-
			R GATTGTCCTGAAGGGTCACC	
il1b	ZDB-GENE-040702-2	*IL1B*	F TGGACTTCGCAGCACAAAATG	(88)
			R GTTCACTTCACGCTCTTGGATG	
cd4-1	ZDB-GENE-100922-280	*CD4*	F TAAAGCACAAGAAAGCCATG	-
			R TACTCTGCGGGTTCCTGTTG	
cd8a	ZDB-GENE-060210-2	*CD8A*	F GGAGTACCAGATCCAGTAACACAC	-
			R AACCTCCGACCAGAGATGTG	
IgM	ZDB-GENE-030925-46	*IGHM*	F AGATCCAATACAAAGATACTATGC	(21)
			R TGGTGAAATGGAATTGTGG	
tbx21	ZDB-GENE-080104-3	*TBX21*	F GGCCTACCAGAATGCAGACA	(27)
			R GGTGCGTACAGCGTGTCATA	
gata3	ZDB-GENE-990415-82	*GATA3*	F GGATGGCACC GGTC ACTATT	(27)
			R CAGCAGACAGCCTCCGTTT	
foxp3a	ZDB-GENE-061116-2	*FOXP3*	F CAAAAGCAGAGTGCCAGTGG	(27)
			R CGCATAAGCAC CGATTCTGC	
ifng1	ZDB-GENE-040629-1	*IFNG*	F CTTTCCAGGCAAGAGTGCAGA	(89)
			R TCAGCTCAAACAAAGCCTTTCG	
il4	ZDB-GENE-100204-1	*IL4*	F CCAGAGTGTGAATGGGATCC	-
			R TTTCCAGTCCCGGTATATGC	

[1] Zebrafish gene names and qPCR primer sequences are listed accompanied with the ZFIN identification codes and the names of the orthologous genes in humans. HGNC, HUGO Gene Nomenclature Committee.

Supplementary References

88. Pressley, M. E., Phelan, P. E.,3rd, Witten, P. E., Mellon, M. T. & Kim, C. H. Pathogenesis and inflammatory response to *Edwardsiella tarda* infection in the zebrafish. *Dev. Comp. Immunol.* **29**, 501-513 (2005).

89. Vojtech, L. N., Sanders, G. E., Conway, C., Ostland, V. & Hansen, J. D. Host immune response and acute disease in a zebrafish model of *Francisella pathogenesis*. *Infect. Immun.* **77**, 914-925 (2009).
